# Triggers of self-focused attention: an ecological momentary assessment study

**DOI:** 10.1186/s13030-023-00273-6

**Published:** 2023-04-23

**Authors:** Mao Nanamori, Nozomi Tomita, Chiaki Kametani, Naomi Matsuda, Hiroaki Kumano

**Affiliations:** 1 Graduate School of Human Sciences, Waseda University, 2-579-15, Mikajima, Tokorozawa, Saitama 359-1192 Japan; 2 Faculty of Human Sciences, Waseda University, 2-579-15, Mikajima, Tokorozawa, Saitama 359-1192 Japan

**Keywords:** Self-focused attention, Social anxiety disorder, Ecological momentary assessment, Trigger

## Abstract

**Background:**

Self-focused attention (SFA) is a major maintenance factor of social anxiety disorder. The two types of SFA, the observer perspective and self-focus on body sensation, increase anxiety in individuals with high levels of social anxiety. However, the triggers of each SFA remain unclear. This study used ecological momentary assessment to identify the factors that elicit SFA in real-life social scenarios.

**Methods:**

The study obtained 316 samples from 22 Japanese university students (4 male:18 female) with high social anxiety who completed momentary measures of stimulus perception and two types of SFA for 10 days. Links to online questionnaires were sent to the participants via e-mails 3 times a day. First, multilevel single regression analyses were used to identify the stimuli that induced the two types of SFA. Between-level interaction with gender was done to determine the effect of gender biasing on the female participants. Next, for the variables that were significantly predictive in these analyses, multilevel multiple regression analyses were conducted with fear of each stimulus as a control variable.

**Results:**

Perception of gaze, evaluation, and authority predicted SFA from the observer perspective. Perception of gaze also predicted self-focus on body sensation. In addition, the perception of positive response and that of stranger predicted self-focus on body sensation depended on gender, implying that the positive response perception of female participants predicted self-focus on body sensation. After controlling for corresponding fear, gaze perception predicted both SFAs, and the perception of authority predicted SFA from the observer perspective. In addition, after controlling for relevant fear, the perception of positive response of female participants predicted self-focus on body sensation. In contrast, the fear of evaluation but not the perception of evaluation predicted SFA from the observer perspective.

**Conclusions:**

The perception of gaze is the most powerful trigger of the two types of SFA, even after controlling for fear of gaze in real-life social scenarios for individuals with social anxiety. SFA from the observer perspective is also triggered by the perception of authority and fear of evaluation. The role of perception of positive responses or strangers should be re-evaluated after correcting for gender imbalance. (350 words / 350 words)

## Background

Social anxiety disorder (SAD) is a psychiatric disorder characterized by marked fear or anxiety regarding social situations in which an individual is exposed to scrutiny from others [1]. SAD displays a continuum of psychological characteristics, such as certain maintenance factors, between patients and individuals with high levels of social anxiety without a SAD diagnosis [2]. Individuals with high levels of social anxiety tend to focus attention on themselves when entering a social situation. This attention bias is identified as self-focused attention (SFA), which plays a crucial role in maintaining social anxiety in cognitive-behavioral models of SAD [3, 4]. The models propose that SFA from the observer perspective, that is, people with social anxiety view themselves through the eyes of others, is important. Taking the observer perspective is associated with increased anxiety [5]. Patients with SAD take the observer perspective when experiencing social situations and when imagining previously experienced social situations [6].

Self-focus on body sensation is also emphasized in cognitive-behavioral models of SAD [3]. For example, individuals with social anxiety estimated their heart rate changes pretty well in social situations, suggesting enhanced awareness of interoceptive information [3]. Participants with SAD exhibited high levels of anxiety and SFA during an experimental manipulation that led them to believe that their heart rate was high during a speech [7]. However, such manipulation of body-state information led to a decrease in the observer perspective [7]. Wells and Papageorgiou [7] suggested that pulse rate manipulation may affect aspects of self-processing associated more directly with private rather than public self-awareness or the content of self-image other than perspective taking. Although awareness of bodily information would lead to SFA, self-focus on body sensation and SFA from the observer perspective may not co-occur. Hass and Eisenstadt [8] indicated that the observer perspective increased when participants did not perceive bodily information but sensed the presence of others. Self-focus on body sensations and SFA from the observer perspective increase independently, and the conditions under which each SFA is enhanced may differ.

The cognitive-behavioral model of Clark and Wells [3] suggests that SFA occurs after perceiving social danger, such as being negatively evaluated by an audience. In a model proposed by Heimberg et al. [4], individuals with social anxiety increase their self-image from the observer perspective after perceiving the presence of the audience. Schultz and Heimberg [9] indicated that both models define social situations in a broad sense such that the specific aspect necessary to trigger an internal shift of attention is unclear. They also indicated that Clark’s conclusion that the initial detection of negative audience behavior can be sufficient to trigger SFA [10] is incompatible with findings related to attentional bias to various threat cues in the environment. For example, individuals with social anxiety display attentional bias to positive faces as well as negative ones [11, 12]. Furthermore, the fear of positive evaluation is important to SAD and predicts anxiety related to social interaction [13], which suggests that the perception of positive behavior can trigger SFA.

In addition to the affective valences of audience behavior, SFA may have some other triggers in a social situation. For example, in a speech task with a social evaluative threat, individuals with high levels of speech anxiety displayed high SFA levels compared with those with low levels of speech anxiety [14]. Specific circumstances of a speech situation, such as being gazed at by the audience, being forced to speak, and being evaluated, could be triggers of SFA. Attention should also change depending on the relationship with others. Interacting with strangers and acquaintances are more associated with social anxiety than interaction with close friend/romantic partners in real life [15]. Individuals with social anxiety experience increased SFA in conversations with unfamiliar people, such as classmates who only greet each other, whereas conversations with familiar people, such as friends who are close to them, lead to decreased SFA [16]. Thus, the perception of acquaintances other than close friends, strangers, and unfamiliar people in social situations may increase SFA and lead to high levels of social anxiety. However, Fujihara [16] did not include strangers in the group of unfamiliar people, and whether or not interactions with strangers increase SFA remains to be unclear. The perception of authority could also lead to internal changes in attention because interacting with authority in a real-life social situation was associated with the highest level of anxiety among different types of interaction partners, such as acquaintances, strangers, close friends/romantic partners, and family [15]. Still, no study has revealed if the perception of authority increases SFA. Although SFA may have possible multiple triggers, as mentioned above in a social situation, the specific triggers of each of the two types of SFA, namely, SFA from the observer perspective and self-focus on body sensation, remain to be clarified.

Previous studies on SFA utilized traditional methods with one-time measures using laboratory designs, such as speech tasks or recalling specific social situations, whereas only a few captured natural social scenarios. The current study used ecological momentary assessment (EMA) to identify the triggers of SFA in multiple natural social situations. EMA is an ecologically valid method that uses a repeated collection of real-time data on the behavior and experience of subjects in their natural environment [17]. Conventional retrospective questionnaire methods present difficulty in collecting accurate and detailed self-reports of experiences in daily life due to extensive memory distortion [18]. In addition, an issue of compliance is common among paper-based retrospective questionnaire methods, which pertains to the possibility that participants are completing momentary reports at a later time instead of according to the protocol [18]. The benefit of using EMA is the collection of self-report data in daily life with minimized recall bias [17]. Recently, EMA studies based on the collection of self-report data can be conducted more easily than previously due to technological advances such as smartphones [19]. Against this background, the present study conducted EMA using smartphones to capture the variability of SFA within an individual in social situations through repeated assessments of daily life. Although Lee [15] conducted an EMA study that investigated the predictive role of perceptions toward an interaction partner on state social anxiety, he did not measure SFA, which is the main target of this study.

The current study had two objectives. The first was to identify the factors that elicit each of the two types of SFA in real-life social situations through the use of EMA. We focused on the perception of various stimuli in social situations as triggers of SFA. We investigated whether SFA would be elicited by perceiving each of nine stimuli: gaze, negative response, positive response, evaluation from other people, speaking environment, and the presence of authority, a stranger, an acquaintance, and a familiar person. The second objective was to identify the triggers that increase SFA according to the perception per se in a social situation. For example, because fear of evaluation is associated with SFA, fear of other stimuli may also be related to SFA by comprising the internal cues of those stimuli. Thus, fear of the nine stimuli was measured as a control variable, where the effect of fear was controlled.

## Methods

### Participants

The participants were recruited from a single university, the one the authors belong to, through an advertisement on a portal site available to all university students. They were screened for social anxiety using the Japanese version of the Liebowitz Social Anxiety Scale (LSAS-J) [20], and the data of 22 (4 male: 18 female) who scored above the boundary score of 30 were included for analysis. The average score of the participants was 72.59 (*SD* = 23.37, minimum = 35, and maximum = 128), whereas the average age was 20.82 years (*SD* = 2.06). All participants were Japanese and reported no psychological disorders, including SAD or traumatic experiences. After the survey, we gave the participants a book voucher worth 1000 JPY.

### Measure of social anxiety (trait measure)

As previously mentioned, the LSAS-J was used to screen for social anxiety. The LSAS-J uses a four-point Likert-type scale (0 = *not at all*; 3 = *totally*) to assess fear and avoidance of 24 common social performance and interaction situations. Scores range from 0 to 144, where the sum of each item is used to rate the severity of social anxiety. The cut-off point of the LSAS-J is 44, whereas the boundary value is 30. The measure has a high internal consistency (*α* = 0.95) and test-retest reliability (0.92). The LSAS-J has been correlated with the Japanese version of the Social Avoidance and Distress scale (SADS: *r* = .65, *p* < .001) [20].

### EMA measures (state measure)

Table 1 displays the questions of EMA.

**Table 1:** Questions of EMA

Questions	How to response
**Social situation**: Reflect on events within the past five hours from now and select the most recent one out of the following social situations you have experienced.	
1. Participating in small groups or meetings	Multiple-choice (select one from ten options)
2. Eating or drinking in public places	
3. Having a one-on-one conversation	
4. Talking on the phone with someone	
5. Acting in front of an audience	
6. Giving a report to a group	
7. Telephoning in public	
8. Working while being observed	
9. Being in a crowded place	
10. I did not experience any social situation	
**Details about the social situation**: Answer the following questions about the social situation you selected in the previous section.	
1. When did that social scene begin and end?	Free form
2. Who were you with in the social situation?	
3. Where were you when experiencing the social situation?	
**Perception of stimuli**: Reflect on the social situation you have experienced, and answer if each of the following items applied to the situation. The expression “the person in the social situation” in the following items includes the person you were calling if the situation was “talking on the phone with someone” or “telephoning in public.“	
1. Gaze from the person in the social situation	2-point scale 1. No 2. Yes
2. Negative responses from the person in the social situation	
3. Positive responses from the person in the social situation	
4. Being evaluated by the person in the social situation	
5. The person in the social situation had more authority than you (e.g., teacher, boss, etc.)	
6. Speaking to or saying something to the person in the situation	
7. The person in the social situation was a stranger	
8. The person in the social situation was an acquaintance	
9. The person in the social situation was close to you (e.g., friends, lover, etc.)	
**Fear of stimuli**: Reflect on the social situation you have experienced, and answer each of the following items to what extent it fitted your fear in the situation.	
1. I was afraid of being looked at by the person in the situation	6-point scale 1. Strongly disagree 2. Disagree 3. Somewhat disagree 4. Somewhat agree 5. Agree 6. Strongly agree
2. I was afraid of negative responses (anger, disgust, etc.) from the person in the situation	
3. I was afraid of positive responses (smile, praise, etc.) from the person in the situation	
4. I was afraid of being evaluated by the person in the situation	
5. I was afraid of authority figures in the situation	
6. I was afraid of speaking to or saying something to the person in the social situation	
7. I was afraid of strangers	
8. I was afraid of acquaintances	
9. I was afraid of familiar persons	
**Self-focused attention from an observer perspective**: Reflect on the social situations you have experienced, and answer each of the following items to what extent it fitted your behavior.	
1. I was worried about myself through other people’s eyes	6-point scale 1. Strongly disagree 2. Disagree 3. Somewhat disagree 4. Somewhat agree 5. Agree 6. Strongly agree
2. I was concerned about how I appeared to others	
3. I imagined how I looked like to others	
4. Although I did not want to worry about how I appeared to others, I couldn’t stop worrying about it	
**Self-focus on body sensation**: Reflect on the social situations you have experienced, and answer each of the following items to what extent it fitted your behavior.	
1. I was paying attention to my complexion	6-point scale 1. Strongly disagree 2. Disagree 3. Somewhat disagree 4. Somewhat agree 5. Agree 6. Strongly agree
2. I was paying attention to my physical reactions (e.g., heart rate)	
3. I was paying attention to my body temperature	
4. I was paying attention to my sweat	

*Social situation.* This question was used to ensure whether the participants experienced certain social situations. The scale included nine representative social situations extracted from the items of the LSAS-J. Four social situations denote interactional communication, such as “participating in small groups or meetings” and “having a one-on-one conversation.” Another four situations pertain to performance, in which people act in front of others, such as “acting in front of an audience” and “working while being observed.” In addition, being in a crowded place is included in the nine social situations. These situations were not only in-person but also online, such as through video conferences. The participants selected one situation that they experienced within 5 hours before answering. They selected the option “I did not experience any social situations” when they had not experienced any of the social situations. After selecting one of the social situations, they were requested to describe the start and end times, the place, and the people with them.

*Perception of stimuli in a social situation.* This question assessed whether the participants perceived nine stimuli (e.g., gaze, negative response, positive response, evaluation, speaking environment, authority, stranger, acquaintance, and familiar person) in the social situations using a two-point scale (0 = no or 1 = yes).

*Fear of stimuli.* This question uses a six-point Likert-type scale (1 = *Strongly disagree*; 6 = *Strongly agree*) to rate nine items and measures of the fear of each stimulus in the social situations.

*SFA from the observer perspective.* This question measures the degree of SFA from the observer perspective in the situation. The items are derived from the Mental Perspective Scale for Social Anxiety Disorder (MPS) [21]. The MPS comprises three subscales: field perspective (MPS-F), observer perspective (MPS-O), and detached mindfulness perspective (MPS-DM). The reliability and validity of the MPS have been reported [21]. We used the MPS-O, which measures how the respondents view themselves through the eyes of others (e.g., I was worried about how I appear to others). The participants rated four items using a six-point Likert-type scale (1 = *Strongly disagree*; 6 = *Strongly agree*).

*Self-focus on body sensation.* This question was modified from the Focused Attention Scale (FAS) [22]. The FAS was developed based on the Focused Attention Questionnaire [23]. The FAS comprises two subscales, namely, FAS-self and FAS-others. FAS-self measures the respondents’ attention to their body sensations (e.g., sweat, heart rate, body temperature, and complexion). FAS-others measures the respondent’s attention to the behavior of others. We used FAS-self to measure self-focus on body sensation. The reliability and validity of the FAS have been reported [22]. The participants rated four items using a six-point Likert-type scale (1 = *Strongly disagree*; 6 = *Strongly agree*).

### EMA procedure

At the first online meeting, the participants were instructed to respond to the questionnaire using smartphones for 10 days. They were sent e-mails with a link to the questionnaire three times per day during the EMA survey: 12:00, 17:00, and 22:00. Five-hour intervals were used to reduce the burden on participants because some social situations in daily life, such as classes or part-time jobs, may last a long time and participants may experience other social situations, such as team meetings, less frequently in a day. The participants were told to respond to the questionnaires within one hour of receiving the e-mail. If an hour passed without their response, they were asked to ignore the prompt and answer the questionnaire in the following e-mail. The questions were related to social situations that the participants experienced within 5 hours before answering. They were instructed that interaction with family members was excluded from social situations, whereas online situations, such as video conferences, were included. They received compensation after the EMA survey.

### Statistical analysis

The EMA data were analyzed through the use of HAD (ver16.03) [24]. The data exhibited a hierarchical structure: repeated assessments (level 1) were nested within individuals (level 2). The hierarchical data contained a combination of level 1 and level 2 variances. Thus, instead of showing descriptive statistics for all the samples, the grand means of the perception of stimuli, the fear of stimuli, and the two types of SFA were calculated, and their between-individual variance and within-individual variance were estimated using multilevel modeling. Next, multilevel modeling was used to examine correlations between each of the perceptions of stimuli. To investigate the first purpose for demonstrating the types of stimuli perceived in a social situation that predicts the two types of SFA, single regression analyses using hierarchical linear modeling (HLM) [25], a multilevel model, were conducted. Although the participants may have experienced several types of stimuli simultaneously, we developed separate models instead of including them in one model because a multiple regression analysis shows the effect of one stimulus while the other stimuli are fixed as their average. Considering the environments of everyday life, a social situation where only one stimulus varies while the other stimuli are controlled does not exist. Therefore, the perception of each of the nine stimuli was used as the independent variable, whereas each of the two types of SFA was used as the dependent variable. Thus, nine analyses each were examined for each dependent variable. The significance level was adjusted with Bonferroni correction set at 0.0056, equal to 0.050 divided by 9. Because there was a gender imbalance, with 18 of the 22 participants being female, we determined its effect by including gender in the interaction term as a level 2 variable in all single regression analyses. Multiple regression analyses using HLM were conducted with fear of stimuli as the control variable to investigate whether the perception per se of stimuli is associated with SFA without related fear, which is the second objective. The variables that significantly predicted SFA by the multilevel single regression analysis were used as the independent variables, fear of those stimuli was used as the control variable, and the two types of SFA were used as the dependent variables. An analysis was conducted for each stimulus. In addition, for variables that had significant interactions with gender, multiple regression analyses were also conducted using fear of the stimulus and gender as control variables. In the analyses, gender was included in the interaction. Level 1 variables were centered by the group means, and Level 2 variables were centered by the grand means before analysis. Shimura et al. [26] conducted a multilevel analysis using 306 EMA data obtained from 62 individuals and modeled the intercept as a random effect and the slope as a fixed effect, considering that the number of data was not sufficient and the stability of the estimation of model coefficients may not be attained. Because we used a similar number of EMA data, 316 data from 22 individuals, we regarded the intercept as a random effect and the slopes of stimuli and fear for them as a fixed value among the participants. All models were estimated using the maximum likelihood method.

## Results

### Number of EMA responses

We sent the participants 660 questionnaires in total, of which 109 were unanswered. Thus, 551 responses were collected in the present EMA survey, and the compliance rate for EMA input was 83.5%. Responses that indicated that the participants experienced no social situations (227 responses; 41.1%), responses describing interaction with family members (3 responses) and responses related to experiences that occurred more than 5 hours before the answer (5 responses), were excluded from the analysis. The final sample included 316 responses.

### Details on EMA data

Table 2 presents the grand means, the between- and within-individual variance of stimulus perception, fear of perception, and SFA estimated by multilevel models in the final samples of EMA. The grand means of perception of stimuli show the frequency rate of reporting among 316 responses. Thus, the proportions of each stimulus perception were as follows: gaze = 73%, negative response = 11%, positive response = 54%, evaluation = 28%, authority = 49%, speaking environment = 69%, stranger = 48%, acquaintance = 38%, and familiar person = 40%. We conducted a multilevel correlation analysis of SFA from the observer perspective and self-focus on body sensation. The two SFAs were moderately, positively correlated (*r* =. 45, *p* < .01).

**Table 2:** The grand mean and between- and within-individual variance of stimulus perception, related fear, and SFA

	Grand mean	Between-individual variance	Within-individual variance
**Perception of stimulus**			
Gaze	0.73	0.02	0.17
Negative response	0.11	0.01	0.09
Positive response	0.54	0.04	0.19
Evaluation	0.28	0.01	0.18
Authority	0.49	0.05	0.18
Speaking environment	0.69	0.03	0.18
Stranger	0.48	0.04	0.20
Acquaintance	0.38	0.03	0.18
Familiar person	0.40	0.06	0.20
**Fear of stimulus**			
Fear of gaze	2.98	0.83	1.81
Fear of negative response	3.47	1.33	2.08
Fear of positive response	1.99	0.73	0.88
Fear of evaluation	2.72	0.94	1.95
Fear of authority	2.04	0.81	1.20
Fear of speaking environment	3.07	0.70	1.92
Fear of stranger	1.79	0.65	1.05
Fear of acquaintance	1.99	0.65	1.33
Fear of familiar person	1.76	0.41	1.18
**Self-focused attention**			
Observer perspective	14.35	14.27	12.62
Self-focus on body sensation	8.20	11.62	8.50

*Note*. The grand means of perception of stimuli shows the frequency rate of reporting among 316 responses

### Reports of social situations

The center three columns of Table 3 illustrate the total, mean, and SD of the number of reports by all participants. Among the nine social situations, having a one-on-one conversation was the most frequent (89 responses), followed by being in a crowded place (78 responses), then by participating in small groups or meetings (66 responses). Giving a report to a group and telephoning in public were the least frequent social situations (five responses). Because each participant gave a different total number of reports and might have shown a different order of experienced social situations, the two right-hand columns of Table 3 illustrate the mean and SD of the percentage of reports for each participant. Among the nine social situations, having a one-on-one conversation was the most frequent (27.7%), followed by being in a crowded place (24.1%), then by participating in small groups or meetings (21.4%). Telephoning in public was the least frequent social situation (1.6%). Therefore, the order from the highest to the lowest in the number of responses by all participants and the order in the means of percentages reported by each participant were almost the same, meaning most participants similarly experienced nine social situations.

**Table 3:** The means and SDs of the number and percentage of reported social situations

Social situations	Number of reports			Percentage of reports	
	Total number of reports	Mean number of reports	SD of the number of reports	Mean of the percentage of reports	SD of the percentage of reports
1. Participating in small groups or meetings	66	3.00	2.43	21.4	15.6
2. Eating or drinking in public places	30	1.36	1.67	9.6	12.1
3. Having a one-on-one conversation	89	4.05	3.61	27.7	22.8
4. Talking on the phone with someone	11	0.50	1.12	3.4	7.0
5. Acting in front of an audience (e.g., giving a talk, performing)	6	0.27	0.62	1.8	4.2
6. Giving a report to a group	5	0.23	0.67	2.0	5.2
7. Telephoning in public	5	0.23	0.67	1.6	4.7
8. Working while being observed	26	1.18	1.30	8.5	9.8
9. Being in a crowded place	78	3.55	2.52	24.1	15.9
Total reports of social situations	316	14.36	4.16	–	–

### The correlations between the nine social stimuli

Table 4 shows the results of the multilevel correlation analyses for the nine social stimuli. There were significant, moderate, positive correlations between authority and acquaintance (*r* = .52, *p* < .010), between positive response and speaking environment (*r* = .49, *p* < .010), and between evaluation and authority (*r* = .45, *p* < .010). A significant, moderate, negative correlation was shown between stranger and familiar person (*r* = − .46, *p* < .010).

**Table 4:** The correlations between the nine social stimuli

Variables	1	2	3		4		5	6		7		8		
1. Gaze	–													
2. Negative response	0.10^†^	–												
3. Positive response	0.37^**^	0.18^*^	–											
4. Evaluation	0.25^**^	0.23^**^	0.35^**^		–									
5. Authority	0.18^**^	0.05	0.24^**^		0.45^**^		–							
6. Speaking environment	0.39^**^	0.22^**^	0.49^**^		0.27^**^		0.28^**^	–						
7. Stranger	− 0.09	− 0.07	− 0.33^**^		− 0.14^*^		− 0.08	− 0.36^**^		–				
8. Acquaintance	0.17^**^	0.02	0.24^**^		0.37^**^		0.52^**^	0.29^**^		− 0.24^**^		–		
9. Familiar person	0.21^**^	0.01	0.37^**^		0.09		0.10^†^	0.38^**^		− 0.46^**^		0.11^†^		

*Note.*^†^*p <* .100, ^*^p < .050, ^**^p < .010

### The relation between perception of each stimulus and each of two types of SFA

Table 5 depicts the results of the single regression analyses using HLM to investigate whether the perception of stimuli predicted the two types of SFA (from the observer perspective and self-focus on body sensation). With Bonferroni correction at significance level set at 0.0056, gaze, evaluation, and authority significantly predicted high levels of SFA from the observer perspective (gaze: *b* (293) *=* 2.10, *p* < .001; evaluation: *b* (293) = 1.67, *p* = .001; authority: *b* (293) = 2.04, *p* = .003). In addition, gaze significantly predicted self-focus on body sensation (*b* (293) = 1.60, *p* < .001).

**Table 5:** The relation between the perception of each stimulus and two types of SFA

	Observer perspective				Self-focus on body sensation		
	*b*	SE	*p*		*b*	SE	*p*
Gaze	2.10	0.49	< 0.001^*^		1.60	0.39	< 0.001^*^
Negative response	0.98	0.65	0.129		0.21	0.44	0.631
Positive response	0.60	0.42	0.155		0.49	0.32	0.122
Evaluation	1.67	0.51	0.001^*^		0.65	0.27	0.015
Authority	2.04	0.68	0.003^*^		0.97	0.43	0.025
Speaking environment	0.95	0.62	0.124		0.17	0.53	0.742
Stranger	0.12	0.48	0.810		0.07	0.48	0.886
Acquaintance	1.51	0.57	0.008		0.42	0.34	0.223
Familiar person	0.28	0.83	0.733		0.29	0.57	0.608

*Note. b*: non-standardized correlation coefficient, SE: standard errors, ^*^p <. 0.0056

### The Relation Between Perception of Each Stimulus and the Two Types of SFA when including gender as a level 2 variable in the interaction

To investigate the effect of gender, we included it in the interaction term as a level 2 variable in the analyses examining the relation between stimuli perception and SFA. Bonferroni with a corrected *p*-value of 0.0056 was used. The results showed that the interaction between a positive response and gender was significant in the model with self-focus on body sensation as the dependent variable (*b* (292) *=* -2.27, *p* < .001), although the main effect of a positive response was not significant. A simple slope analysis showed that the main effect was significant for both the female and male groups (female: *b* (292) *=* 0.91, *p* = .001, male: *b* (292) *=* -1.36, *p* < .001), which means SFA was increased in the female group by perceiving positive response, and vice versa was found for the male group. The interaction between stranger and gender was also significant in the model with self-focus on body sensation as the dependent variable (*b* (292) *=* 3.04, *p* = .004), although the main effect of stranger was not significant. A simple slope analysis showed that the main effect was marginally significant for the male group (*b* (292) *=* 2.59, *p* = .006). The interaction between authority and gender showed a marginally significant *p*-value of 0.008 (*b* (292) = -3.09). A simple slope analysis showed that the main effect was significant in the female group (*b* (292) = 2.49, *p* < .001).

### Correlations coefficient for perception of stimulus and fear of each stimulus

Multilevel correlation analyses between the stimuli that significantly predicted SFA, shown in Table 5, and related fear were conducted to examine multicollinearity problems in multiple regression analyses that used HLM. Gaze, evaluation, and authority, which significantly predicted SFA, were examined for the correlation with their fearful affect. They all displayed low significant correlations between their perceptions and corresponding feelings of fear (gaze and fear of gaze: *r* = .20, *p* < .010; evaluation and fear of evaluation: *r* = .34, *p* < .010; and authority and fear of authority: *r* = .23, *p* < .010). In addition, positive response and stranger, which had significant interactions with gender, were examined for the correlation with their fearful affect. They both displayed low significant correlations with their corresponding feelings of fear (positive response and fear of positive response: *r* = .05, *n.s.*; stranger and fear of stranger: *r* = .09, *n.s.*). Thus, we regarded multicollinearity as not being a problem for the multiple regression analyses.

### Multiple regression analysis of the perception of each stimulus and each of two types of SFA, with fear of each stimulus as control variables

The upper three variables of Table 6 represent the results of the multiple regression analyses using HLM for the stimuli that significantly predicted SFA in Table 5 after controlling for fear of corresponding stimuli. Concerning SFA from the observer perspective, the significance level was adjusted with Bonferroni correction at 0.017, equal to 0.050 divided by 3, because it was significantly related to gaze, authority, and evaluation. For self-focus on body sensation the significance level was also adjusted at 0.017 because it was related to gaze and interactions of gender with positive response and stranger. First, gaze was analyzed as the independent variable, SFA from the observer perspective was considered the dependent variable, and fear of stimulus was used as the control variable. The results demonstrated that gaze significantly predicted SFA from the observer perspective (*b* (292) = 1.47, *p* < .001). Second, the results for evaluation demonstrated that it did not predict SFA (*b* (292) = 0.68, *p* = .074), while fear of evaluation did predict SFA (*b* (294) = 0.90, *p* < .001). Third, the results for authority demonstrated that it significantly predicted SFA from the observer perspective after controlling for relevant fear (*b* (292) = 1.83, *p* = .004). Fourth, gaze was analyzed as the independent variable, self-focus on body sensation was designated as the dependent variable, and related fear was used as the control variable. The results illustrated that gaze perception significantly predicted self-focus on body sensation (*b* (292) = 1.30, *p* = .001).

**Table 6:** The relation between the perception of stimuli and SFA when controlling for fear of stimuli

	Observer perspective				Self-focus on body sensation		
	*b*	SE	*p*		*b*	SE	*p*
**Variables 1**							
Gaze	1.47	0.41	< 0.001^*^		1.30	0.40	0.001^*^
Fear of gaze	1.01	0.29	0.001^*^		0.49	0.24	0.039
**Variables 2**							
Evaluation	0.68	0.38	0.074		–	–	–
Fear of evaluation	0.90	0.19	< 0.001^*^		–	–	–
**Variables 3**							
Authority	1.83	0.64	0.004^*^		–	–	–
Fear of authority	0.37	0.28	0.198		–	–	–
**Variables 4**							
Positive response × gender	–	–	–		-2.18	0.40	< 0.001^*^
Fear of positive response	–	–	–		0.45	1.67	0.007^*^
**Variables 5**							
Stranger × gender	–	–	–		3.02	1.11	0.007^*^
Fear of stranger	–	–	–		0.45	0.20	0.023

*Note. b*: non-standardized regression coefficients, SE: standard errors, ^*^p < .017

### Multiple regression analysis of the perception of each stimulus and self-focus on body sensation with fear of each stimulus and gender as control variables

The lower two variables of Table 6 present the results of the multiple regression analyses using HLM for the stimuli that indicated significant interaction with gender in the model with self-focus on body sensation as the dependent variable after controlling for fear of corresponding stimuli. Gender was included in the interaction as a level 2 variable, and Bonferroni corrected *p*-value was 0.017. The interaction between gender and positive response was significant (*b* (292) = -2.18, *p* < .001), while the main effect of positive response was not. A simple slope analysis showed that the main effect was significant for both the female and male groups (female: *b* (292) *=* 0.85, *p* = .002, male: *b* (292) *=* -1.33, *p* < .001), which means SFA increased in the female group by perceiving positive response, and vice versa was seen in the male group. The interaction between gender and stranger was significant (*b* (292) = 3.02, *p* = .007), while the main effect of stranger was not. A simple slope analysis showed that the main effect was significant for the male group (*b* (292) *=* 2.48, *p* = .015). These results were almost the same as shown in the analyses without controlling for corresponding feelings of fear.

## Discussion

This study used EMA to capture the perception of stimuli and two types of SFA in real-life social situations. Individuals with high levels of social anxiety displayed increased SFA from the observer perspective when perceiving gaze, evaluation, and authority in everyday social situations. They also displayed increased self-focus on body sensation when perceiving gaze. In addition, for positive response and stranger, there was a gender effect in predicting self-focus on body sensation. Considering the scarcity of male participants, the significant result for positive response and the marginally significant result for stranger in the male group should be interpreted cautiously. Still, it can be said that the female group displayed increased self-focus on body sensation when perceiving positive responses. The perception of gaze increased both types of SFA, even when the perception of stimuli was controlled for fear of gaze. Thus, the perception of gaze would be a strong candidate for the specific aspect of social situations necessary to trigger an internal shift of attention reported by Schultz and Heimberg [9].

The observer perspective should be triggered by perceiving the presence of an audience [4]. Therefore, the perception of audiences should consist of gaze, evaluation, or authority for individuals with high levels of social anxiety. The perception of gaze leads to the perception of existing audiences [27], and eye contact enhances public self-awareness, which is the feeling of how one is perceived by others [28]. However, previous studies have never evaluated the role of gaze perception in predicting SFA in individuals with social anxiety, and this study demonstrates this connection for the first time. Mansell et al. [14] found that people with high levels of anxiety for a speech displayed higher levels of SFA in a speech situation in which they believed audiences would evaluate them, which was similar to the present result. If an authoritative person is a member of the audience, anxiety may increase the most [15], but an impact on SFA was not shown, and the present result was the first to show this impact. Women have been reported to experience greater fear than men when speaking with authority Fig. [29]. In the present study, the interaction between authority and gender was marginally significant, and the main effect of authority was significant in the female group in the simple slope analysis. Thus, the auxiliary results of the present study corroborated the previous studies. In addition, the perception of gaze also predicted self-focus on body sensation. The result indicated that it was a common eliciting factor for both types of SFA, although the two SFAs were qualitatively different. However, other factors, such as evaluation and authority, did not significantly predict self-focus on body sensation.

In the current study, the perception of positive response predicted self-focus on body sensation in the female participants, which was maintained even after controlling for corresponding fear. In addition, the perception of evaluation did not predict self-focus on body sensation, which means that the perception of positive response per se, not related to fear or being positively evaluated, affected the abovementioned result. Socially anxious individuals tend to interpret positive social events in a way that maintains a sense of social threat [30]. Furthermore, female adolescents interpret ambiguous events more negatively than males of the same age [31]. Thus, females may be more likely to pay attention to body sensations such as blushing and body temperature by interpreting positive responses more negatively in daily social situations.

The finding that the perception of negative response from others did not predict either type of SFA was inconsistent with the models of Clark and Wells [3] and Heimberg et al. [4]. This result was also inconsistent with that of Veljaca and Rapee [32], who demonstrated that people with high levels of SFA were more likely to detect negative behavior. As Schultz and Heimberg [9] indicated, the models mentioned above broadly define social situations, and the present results’ inconsistency may be due to that vague definition. In the present study, the perception of negative and positive responses per se did not predict SFA from the observer perspective, but the evaluation accompanying those perceptions did predict SFA from the observer perspective. That was consistent with Mansell et al. [14], who found that people with speech anxiety showed higher levels of SFA when they believed audiences would evaluate them. Therefore, these results showed that evaluation might be a more relevant aspect of social situations necessary to trigger an internal shift of attention to SFA from observer perspective rather than negative and positive responses.

The speaking environment did not significantly predict either type of SFA. This factor differed from others in that it may indicate not the perception of but participation in such an environment. Although it did not significantly predict either type of SFA, the coefficient was greater for SFA from the observer perspective than for self-focus on body sensation. This may denote a difference between the two types of SFA. The perceptions of acquaintance and familiar person were also not relevant to either SFA. The results suggest that closeness to the interaction partner is not associated with SFA. In the current study, SFA may be related to whether the interaction partner is higher in social status, such as in a position of authority, than oneself in the situation rather than whether the partner is close or not.

Results related to the second objective indicated that only the perception of gaze increased both types of SFA after controlling for relevant fear. Patients with SAD exhibit abnormal gaze perception compared with control subjects [33]. Using “the cone of gaze” paradigm that required observers to adjust the eyes of a virtual head to the margins of the area of mutual gaze, Gamer et al. [34] found that cone of gaze was widened in SAD patients when a second head also looking at the subject was present. A face-to-face situation was used by Honma [35], who found that the perceptual volume is much larger than the actual volume of eye contact and that the perceptual volume and social anxiety traits were positively correlated. Thus, patients with SAD may perceive gaze even if nobody sees them in a social situation. The current EMA study was unable to measure whether gaze was actually present in social situations, and it should also detect a misperception or an overperception of gaze. Nonetheless, the feeling of being watched has a strong effect on behavior, and such a feeling can be induced without direct-gaze cues [36]. If misperception or overperception of gaze leads to increased SFA, then appropriating gaze perception would be necessary for reducing SFA for individuals with high levels of social anxiety. Harbort et al. [37] demonstrated that a cognitive behavioral therapy program could narrow the gaze cones of patients with SAD. Thus, SFA would decrease more by incorporating sessions that encourage appropriate gaze perception in real-life social situations and assessing it before and after these sessions. For instance, manipulating attention to be well-balanced with external social environments, such as situational attentional refocusing (SAR) [38], could reduce SFA and anxiety. SAR is applied as a means of disrupting unhelpful attention patterns that maintain an unrealistic sense of threat [39]. Thus, for example, telling individuals with SAD to pay attention to the gaze directed by others and to confirm if others are really looking at them in SAR may help to promote SFA reduction. The perception of authority also significantly predicted SFA from the observer perspective after controlling for fear of them. This finding indicated that when individuals with social anxiety interact with figures of authority, they may experience increased SFA from the observer perspective despite the lack of fear of them. These results indicated that SFA from the observer perspective is more sensitive to the perception of relevant persons than does self-focus on body sensation.

The perception of evaluation did not predict SFA from the observer perspective after controlling for its fear, but that fear significantly predicted the SFA. Thus, it was not the evaluation accompanying negative and positive responses that predicted SFA from the observer perspective, but it was the fear of that evaluation that predicted the SFA. Both the fear of negative evaluation and that of positive evaluation are claimed as strong predictors of SAD [13, 40], and the present results are consistent with those contentions. Thus, in addition to the perception of gaze and authority, the fear of evaluation may be one more candidate for social situations that trigger an internal shift of attention to SFA from observer perspective.

### Limitations and future studies

The current study has several limitations, and we suggest future directions to address these limitations. First, although the time periods were relatively short, there is still the possibility of recall bias. The participants had to recall a situation they had experienced a few hours previously, and the process of recalling a social situation might distort or generalize somewhat the content of memory. Therefore, future studies should be designed to have participants report during or immediately after experiencing social situations to minimize recall bias. Second, this study used only subjective reports. We could not objectively measure the details of the actual social situation because the present EMA study measured variables according to participant responses through smartphones. As previously mentioned, patients with SAD exhibit abnormal gaze perception. Thus, the participants may have responded that they perceived gaze from others even when no one gazed at them in social situations. In addition, the other stimuli might also be overreported or underreported. Thus, future studies should manipulate stimuli, including gaze from others in a social situation, and should investigate the relation between the presence of stimuli, subjective perception, and SFA. Liao et al. [41] developed an interactive virtual reality speech simulation system that autonomous audiences provide real-time feedback on the speaker’s behavior. To manipulate stimuli while maintaining ecological validity, future studies should use such a VR system, which would allow us to manipulate variables in social situations that closely resemble real-life experiences. Third, the participants were Japanese university students with high social anxiety but were not SAD patients. SAD patients and individuals with high social anxiety may differ in the context of social situations they experience and the triggers of their SFA. Individuals with high social anxiety exhibit a negative interpretation bias during SFA in social situations [42], and the degree of that bias is related to the severity of SAD [43]. Thus, if SAD patients are targeted, the perception of negative response of others per se may trigger SFA by interpreting the reactions more negatively. Therefore, future studies should examine the triggers of SFA in patients with SAD. Furthermore, we only measured the perception of several stimuli and SFA in social situations specific to university students, such as classes, part-time jobs, and club activities. There may also be cultural differences in the obtained results. Future studies should recruit participants with a broader age range, including employed individuals and participants from other countries, and capture various social situations. Fourth, although the gender effect was incorporated as an interaction term, the present results for men were only preliminary because 18 of the 22 participants were women. Future studies need to include more male participants and be analyzed according to gender. Fifth, we did not screen for autism spectrum disorder (ASD). Steensel et al. [44] showed that 16.6% of young people with ASD have a comorbid social anxiety disorder. In other words, SAD is common among youth with ASD. Brain mechanisms during gaze perception differ between individuals with and without ASD [45]. Future studies need to clarify the differences in cognitive processing, such as stimulus perception and SFA, between people with SAD with and without ASD. Sixth, the current study could not distinguish between in-person and online social situations because the distinction was not asked. However, a previous study found that individuals with high social anxiety showed lower social anxiety during online interactions than in real-life ones [46]. In addition, the perception of stimuli and the degree of SFA may also differ. Therefore, future studies should take into consideration the two situation types.

## Conclusions

This was the first study to use EMA to explore the perception of a wide variety of real-life social situations eliciting SFA. The results demonstrated that the perception of gaze was a common trigger for SFA, both from the observer perspective and body sensation, while the perception of evaluation and authority were also triggers for SFA from the observer perspective. The perception of positive response was also a trigger for the self-focus on body sensations of female participants. Furthermore, gaze perception was relevant to both types after controlling for fear of gaze.

## Data Availability

The datasets used or analyzed during the current study are available from the corresponding author upon reasonable request.

## Abbreviations

SFA: Self-focused attention
SAD: Social anxiety disorder
EMA: Ecological momentary assessment
LSAS-J: the Japanese version of the Liebowitz Social Anxiety Scale
MPS: the Mental Perspective Scale for Social Anxiety Disorder
FAS: the Focused Attention Scale
HLM: Hierarchical linear modeling
SAR: Situational Attentional Refocusing
ASD: Autism spectrum disorder
